# Novel VHH-Based Tracers with Variable Plasma Half-Lives for Imaging of CAIX-Expressing Hypoxic Tumor Cells

**DOI:** 10.1021/acs.molpharmaceut.1c00841

**Published:** 2022-01-19

**Authors:** Sanne A.M. van Lith, Fokko J. Huizing, Gerben M. Franssen, Bianca A.W. Hoeben, Jasper Lok, Sofia Doulkeridou, Otto C. Boerman, Martin Gotthardt, Paul M.P. van Bergen en Henegouwen, Johan Bussink, Sandra Heskamp

**Affiliations:** † Department of Medical Imaging, Radboud University Medical Center, Nijmegen 6500 HB, The Netherlands; ‡ Department of Radiation Oncology, Radboud University Medical Center, Nijmegen 6500 HB, The Netherlands; § Department of Radiation Oncology, University Medical Center Utrecht, Utrecht 3508 GA, The Netherlands; ∥ Department of Cell Biology, University of Utrecht, Utrecht, 3584 GE, The Netherlands

**Keywords:** variable domain of heavy chain only antibody (VHH), tumor hypoxia, carbonic anhydrase IX (CAIX), albumin-binding domain (ABD)

## Abstract

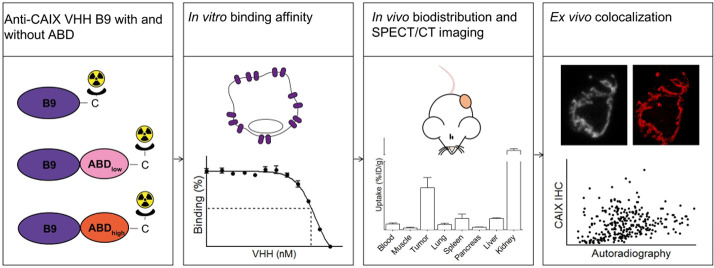

Hypoxic areas are present in the majority of solid tumors, and hypoxia is associated with resistance to therapies and poor outcomes. A transmembrane protein that is upregulated by tumor cells that have adapted to hypoxic conditions is carbonic anhydrase IX (CAIX). Therefore, noninvasive imaging of CAIX could be of prognostic value, and it could steer treatment strategies. The aim of this study was to compare variants of CAIX-binding VHH B9, with and without a C-terminal albumin-binding domain with varying affinity (ABD_low_ and ABD_high_), for SPECT imaging of CAIX expression. The binding affinity and internalization of the various B9-variants were analyzed using SK-RC-52 cells. Biodistribution studies were performed in mice with subcutaneous SCCNij153 human head and neck cancer xenografts. Tracer uptake was determined by *ex vivo* radioactivity counting and visualized by SPECT/CT imaging. Furthermore, autoradiography images of tumor sections were spatially correlated with CAIX immunohistochemistry. B9-variants demonstrated a similar moderate affinity for CAIX *in vitro*. Maximal tumor uptake and acceptable tumor-to-blood ratios were found in the SCCNij153 model at 4 h post injection for [^111^In]In-DTPA-B9 (0.51 ± 0.08%ID/g and 8.1 ± 0.85, respectively), 24 h post injection for [^111^In]In-DTPA-B9-ABD_low_ (2.39 ± 0.44%ID/g and 3.66 ± 0.81, respectively) and at 72 h post injection for [^111^In]In-DTPA-B9-ABD_high_ (8.7 ± 1.34%ID/g and 2.43 ± 0.15, respectively)_._ An excess of unlabeled monoclonal anti-CAIX antibody efficiently inhibited tumor uptake of [^111^In]In-DTPA-B9, while only a partial reduction of [^111^In]In-DTPA-B9-ABD_low_ and [^111^In]In-DTPA-B9-ABD_high_ uptake was found. Immunohistochemistry and autoradiography images showed colocalization of all B9-variants with CAIX expression; however, [^111^In]In-DTPA-B9-ABD_low_ and [^111^In]In-DTPA-B9-ABD_high_ also accumulated in non-CAIX expressing regions. Tumor uptake of [^111^In]In-DTPA-B9-ABD_low_ and [^111^In]In-DTPA-B9-ABD_high_, but not of [^111^In]In-DTPA-B9, could be visualized with SPECT/CT imaging. In conclusion, [^111^In]In-DTPA-B9 has a high affinity to CAIX and shows specific targeting to CAIX in head and neck cancer xenografts. The addition of ABD prolonged plasma half-life, increased tumor uptake, and enabled SPECT/CT imaging. This uptake was, however, partly CAIX- independent, precluding the ABD-tracers for use in hypoxia quantification in this tumor type.

## Introduction

Hypoxia is a key feature of solid tumors. As it is closely associated with disease progression and therapy resistance,^1,2^ identification of hypoxic subvolumes through noninvasive imaging could aid in steering cancer treatment. Therefore, the development of imaging probes detecting hypoxia is ongoing. Traditional probes like [^18^F]-FMISO, [^18^F]-FAZA and [^18^F]-HX4 accumulate in tissues with oxygen pressures below 10 mmHg at any point in time during circulation of the probe.^3−5^ Therefore, they indicate diffusion-limited hypoxia and tumor areas with longer-lasting perfusion-limited hypoxia, while, for treatment resistance, especially the cells that have adapted to hypoxic conditions are of interest. Carbonic anhydrase IX (CAIX) is a transmembrane protein that is upregulated on tumor cells that have adapted to hypoxic conditions,^6,7^ and consequently, imaging of CAIX is a potential strategy to visualize clinically relevant hypoxia.

The variable domains of heavy chain only antibodies (VHHs) are biomolecules with high potential for tumor imaging and therapy.^8−12^ Due to their low molecular weight of about 15 kDa, VHHs are rapidly cleared from the blood through glomerular filtration, leading to high tumor-to-background ratios at early time points after injection. Furthermore, the small size of VHHs leads to efficient and homogeneous tissue penetration.^13,14^ Though fast blood clearance is advantageous for image quality at early time points after injection, it can also limit the absolute uptake of the VHH in tumor lesions, which is problematic for imaging of low-abundant targets or in therapeutic applications, for which high absolute uptake of the drug is important.^15^ To this end, strategies to increase plasma half-life have been applied to VHHs, such as the addition of polyethylene glycol (PEG), conjugation to the Fc domain of conventional antibodies, and engineering of multimeric or multivalent VHH compounds.^16−18^ Another promising method to extend the plasma half-life of compounds is the addition of an albumin-binding domain (ABD).^19^ Albumin is an abundant serum protein with a plasma half-life of 19 days, which is continuously recycled via neonatal F_C_ receptor (FcRn)-mediated transcytosis. Association of the VHH with albumin will thus lead to prolonged serum residence through these mechanisms.^18,20^ In previous studies, this strategy has been shown to increase plasma half-lives of other imaging and therapy tracers such as peptides, VHHs and affibodies.^21−30^

Here, we used the previously selected VHH against CAIX.^31^ The radiolabeled VHH B9 was further developed and characterized for nuclear imaging of CAIX. We fused B9 to albumin-binding domains with varying affinity (ABD_low_ and ABD_high_),^26^ to assess the effect on the pharmacokinetics of the tracer and tumor uptake. We validated the potential of these tracers for nuclear imaging of endogenous hypoxic CAIX-expressing regions in a human head and neck squamous cell carcinoma xenograft model.

## Materials and Methods

### VHH Expression and Conjugation

Anti-CAIX VHH B9^31^ and B9 with a C-terminal albumin-binding domain (ABD) of streptococcal protein G^26^ were used in this study. The VHHs were recloned to vectors that introduce a C-terminal cysteine, followed by the FLAG tag for B9 and the EPEA tag for B9-ABD_high_ and B9-ABD_low,_ and produced as described previously.^31^ In short, bacteria were grown in Terrific Broth (TB, for B9) or 2× Trypton Yeast Extract medium (2×TY, B9-ABD_low_ and B9-ABD_high_) and VHH production was induced with 1 mM Isopropyl β-d-1-thiogalactopyranoside (IPTG) overnight at 25 °C. B9 was purified from the periplasmic fraction using a HiTrap protein A HP column (GE Healthcare, Chicago, IL, USA), and B9-ABD_low_ and B9-ABD_high_ were purified with a HiTrap protein A HP column and a CaptureSelect C-tag Prepacked Column (Thermo Scientific, Waltham, MA, USA). To reduce the cysteines, VHHs were incubated with Tris(2-carboxyethyl)phosphine hydrochloride (TCEP; Sigma-Aldrich, Saint Louis, MS, USA) at a molar ratio of 1:2.75 overnight at room temperature in PBS pH 7.5. Subsequently, VHHs were incubated with maleimide-DTPA (C-107; Chematech, Dijon, France) at a molar ratio of 1:5 in PBS pH 7.5 for 3 h at room temperature. The reaction mixture was dialyzed in 0.25 M ammonium acetate buffer, pH 5.5, to remove excess maleimide-DTPA.

### Binding to Murine and Human Albumin

Human serum albumin (HSA) and mouse serum albumin (MSA) (Sigma-Aldrich BV, Zwijndrecht, The Netherlands) were coated on MaxiSorp plates (5 μg/well) and blocked with 1% BSA/PBS. Upon incubation with 0.5–1000 nM of each VHH in 1% BSA/PBS for 2 h at room temperature, the unbound fraction was washed away, and the bound VHHs were detected with a combination of a rabbit anti-VHH antibody (clone QE19, QVQ B.V.) and a goat anti-Rabbit IRDye800CW (LI-COR Biosciences). The signal was detected using the Odyssey Infrared Imager (LI-COR Biosciences), and fluorescent intensities were plotted over VHH concentration. The binding affinity (*K*_D_) was determined by a nonlinear regression curve fitting for one-site specific binding.

### Radiolabeling and Quality Control

DTPA-B9, DTPA-B9-ABD_low_, and DTPA-B9-ABD_high_ were incubated with ^111^InCl_3_ (Curium, Petten, The Netherlands) in 0.5 M 2-(*N*-morpholino)ethanesulfonic acid (MES) buffer, pH 5.5, for 30 min at room temperature (0.37 MBq/μg for the binding and IC_50_ assay, 0.037 MBq/μg for the Scatchard assay and the indicated specific activity for *in vivo* experiments). Labeling efficiency and radiochemical purity were determined by instant thin-layer chromatography (ITLC) on a silica gel chromatography strip (Biodex, Shirley, NY, USA), using 0.1 M citrate buffer, pH 6.0 as the mobile phase. Purity exceeded 95% in all experiments.

### Cell Culture

SK-RC-52 (clear cell renal cell carcinoma cell line which ubiquitously overexpresses CAIX independent of oxygenation status)^32^ was cultured in RPMI-1640 (GIBCO, Thermo-Fisher Scientific, Waltham, MA, USA), supplemented with 2 mmol/L glutamine (GIBCO) and 10%FCS (Sigma-Aldrich, Saint Louis, MS, USA) at 37 °C in a humidified atmosphere with 5% CO_2_.

### Binding and Internalization Assay

SK-RC-52 cells were grown to confluency (>90%) in 6-well plates and incubated with 1600 Bq [^111^In]In-DTPA-B9, [^111^In]In-DTPA-B9-ABD_low_, or [^111^In]In-DTPA-B9-ABD_high_ in 0.5% BSA in RPMI (binding buffer) for 1, 2, 24, and 48 h. The competition was performed by coincubation with 1 μg of unlabeled B9 or 30 μg of unlabeled girentuximab per well. Cells were washed twice with binding buffer, and receptor-bound VHH was collected by incubation with 0.1 M acetic acid and 154 mM NaCl, pH 2.6, for 10 min at 4 °C. After washing with binding buffer, cells were collected with 1 mL of 0.1 M NaOH. Activity in both receptor-bound and internalized fractions were counted in a γ-counter (2480 Wizard 3′′, LKB/Wallace, PerkinElmer, Boston, MA, USA).

### IC_50_

SK-RC-52 cells were grown to confluency in 6-well plates and incubated with increasing concentrations (0.005–300 nM) of either unlabeled DTPA-B9, DTPA-B9-ABD_low_, or DTPA-B9-ABD_high_ in binding buffer, in the presence of 1600 Bq of [^111^In]In-DTPA-B9 for 4 h on ice. Upon washing, cell fractions were collected using 1 mL of 0.1 M NaOH, and activity was counted in a γ-counter. The IC_50_ values (the concentrations that are required to inhibit binding of [^111^In]In-DTPA-B9 with 50%) were determined in GraphPad Prism.

### Scatchard Assay

SK-RC-52 cells were grown to confluency in 6-well plates and incubated with increasing concentrations of [^111^In]In-DTPA-B9, [^111^In]In-DTPA-B9-ABD_low_, or [^111^In]In-DTPA-B9-ABD_high_ (3–3000 pM) in binding buffer for 4 h on ice. The specificity of binding was determined by coincubation with 1 μg unlabeled B9 per well. After incubation, cells were washed twice with PBS and collected with 1 mL of 0.1 M NaOH. Activity was counted in a γ-counter. The dissociation constant (*K*_d_) was determined in GraphPad Prism.

### Tumor Model

The patient-derived head and neck squamous cell carcinoma (HNSCC) xenograft model SCCNij153^33^ was used for *in vivo* experiments. Six to eight week old female athymic BALB/c nu/nu mice (Janvier Laboratories, Le Genes-Saint-Ile, France) were implanted subcutaneously with a tumor (2 mm diameter) on the right hind leg. Animals were included when tumors were >80 mm^3^ (4–5 weeks after implantation). Allocation to the treatment groups was block-randomized by tumor size. The studies were approved by the Central Authority for Scientific Procedures on Animals (RU-DEC-2015-0071) and carried out under the supervision of the local Animal Welfare Body. Note that ethical approval for the mice experiments in this study was provided on September 8, 2015 by the institutional Animal Welfare Committee of the Radboud University Medical Center (application no. AVD103002015209), in accordance with the guidelines of the Revised Dutch Act on animal experimentation.

### *In Vivo* Dose Escalation

Tumor uptake of [^111^In]In-DTPA-B9 was determined in a dose-escalation study in mice bearing SCCNij153 xenografts. Mice (*n* = 3–5/group) were injected intravenously with 1, 5, or 25 μg of 1 MBq [^111^In]In-DTPA-B9 in PBS/0.5%BSA. A separate group of mice (*n* = 3) was injected with 300 μg unlabeled B9 at 3 h before injection of 5 μg of 1 MBq [^111^In]In-DTPA-B9 to determine the specificity of uptake. Mice were euthanized by CO_2_/O_2_ asphyxation at 4 h after tracer injection. Tumors and other tissue samples were harvested and weighed, and radioactivity in these samples was determined in a γ-counter. Radioactivity concentrations were calculated as a percentage of the injected dose per gram of tissue (%ID/g).

### *In Vivo* Comparison of [^111^In]In-DTPA-B9, [^111^In]In-DTPA-B9-ABD_low_, and [^111^In]In-DTPA-B9-ABD_high_

Mice bearing SSCNij153 xenografts were injected intravenously with 0.33 nmol of [^111^In]In-DTPA-B9 (5 μg), [^111^In]In-DTPA-B9-ABD_low_ (7.4 μg), or [^111^In]In-DTPA-B9-ABD_high_ (7.1 μg) (1 MBq per animal, *n* = 4–5 mice per group). Mice were euthanized by CO_2_/O_2_ suffocation at either 4, 24, or 72 h after tracer injection. Tumors and other tissue samples were used for radioactivity counting as described above.

### MicroSPECT Imaging

Mice underwent microSPECT imaging (U SPECT-II; MILabs, Utrecht, The Netherlands) at 4, 24, or 72 h after injection of 10 MBq 0.33 nmol [^111^In]In-DTPA-B9 (5 μg, *n* = 3), [^111^In]In-DTPA-B9_low_ (7.4 μg, *n* = 3), or [^111^In]In-DTPA-B9_high_ (7.1 μg, *n* = 3), respectively. For each tracer, 3 mice were injected with an excess of monoclonal anti-CAIX antibody girentuximab (1 mg/mouse) 24 h before tracer injection to determine CAIX-specificity of uptake. Mice were anesthetized with isoflurane, and the tumor area was scanned in the prone position using the 1.0 mm diameter multipinhole mouse collimator. SPECT scans were acquired for 120 min using 6 bed positions. After scanning, mice were injected intravenously with Hoechst 33342 (15 mg/kg, Sigma, Zwijndrecht, The Netherlands), and after 1 min, they were euthanized by cervical dislocation. Tissue samples and half of the tumors were used for radioactivity counting, and the other half of the tumors were snap-frozen in liquid nitrogen for immunohistochemistry and autoradiography as described.

Scans were reconstructed with 4 iterations and 16 subsets using an ordered-expectation maximization algorithm with a voxel size of 0.375 mm (MILabs reconstruction software). SPECT images were analyzed with Inveon Research Workplace software (version 3.0; Siemens Preclinical Solutions).

### Autoradiography and CAIX Immunohistochemistry

Snap-frozen tumors were cut into 5 μm sections and mounted on poly l-lysine coated slides. These were exposed to phospholuminescence plates in a Fujifilm BAS cassette 2025 for 36 h (Fuji Photo Film), and scanned using a Fuji BAS-1900 II bioimaging analyzer at a pixel size of 25 μm × 25 μm. After fixation with cold acetone for 10 min at 4 °C, immunohistochemical staining and analysis for CAIX and Hoechst was performed as described previously.^34^ Intratumoral correlation analysis was performed as described by Huizing et al.^35^ by resizing and aligning CAIX immunohistochemistry and autoradiography images. A parametric mapping technique was applied, and a correlation coefficient for colocalization of CAIX staining and autoradiography in viable tumor tissue was calculated.

### Statistics

Graphpad Prism was used for statistical analyses. Statistical significance was determined with an unpaired *t* test or one-way ANOVA, and correlation coefficients were calculated with Pearson tests. *P* values ≤ 0.05 were considered significant. * = *p* ≤ 0.05, ** = *p* ≤ 0.01, and *** = *p* ≤ 0.001.

## Results

### Affinity of the B9-Based Tracers for Human and Murine Serum Albumin

**Figure 1:**
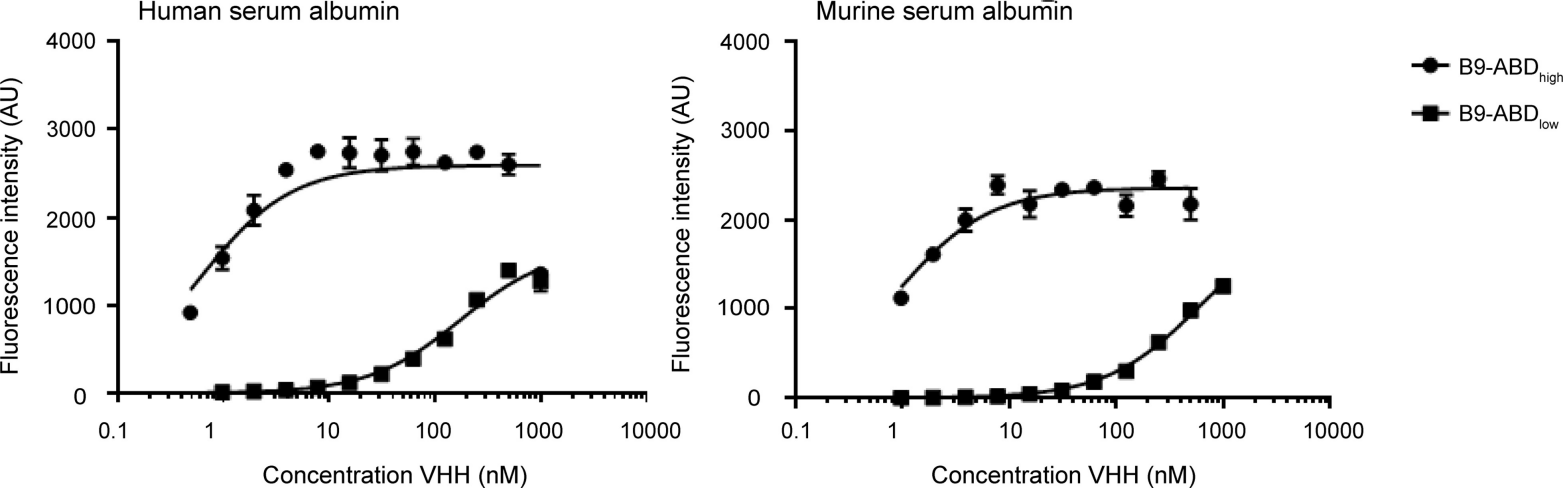
Binding of various concentrations of fluorescently labeled B9-ABD_low_ and B9-ABD_high_ to coated human and murine serum albumin to determine binding affinity.

B9, B9-ABD_low_, and B9-ABD_high_ were successfully produced, and binding of the ABD to MSA and HSA was verified *in vitro* (Figure 1). As expected, binding affinity to HSA and MSA was higher for B9-ABD_high_ (*K*_D_ = 0.59 nM and 0.88 nM, respectively) as compared to B9-ABD_low_ (*K*_D_ = 173.4 nM and 579.4 nM, respectively).

### [^111^In]In-DTPA-B9, [^111^In]In-DTPA-B9-ABD_low_, and [^111^In]In-DTPA-B9-ABD_high_ Bind to CAIX Expressing Cells

**Figure 2:**
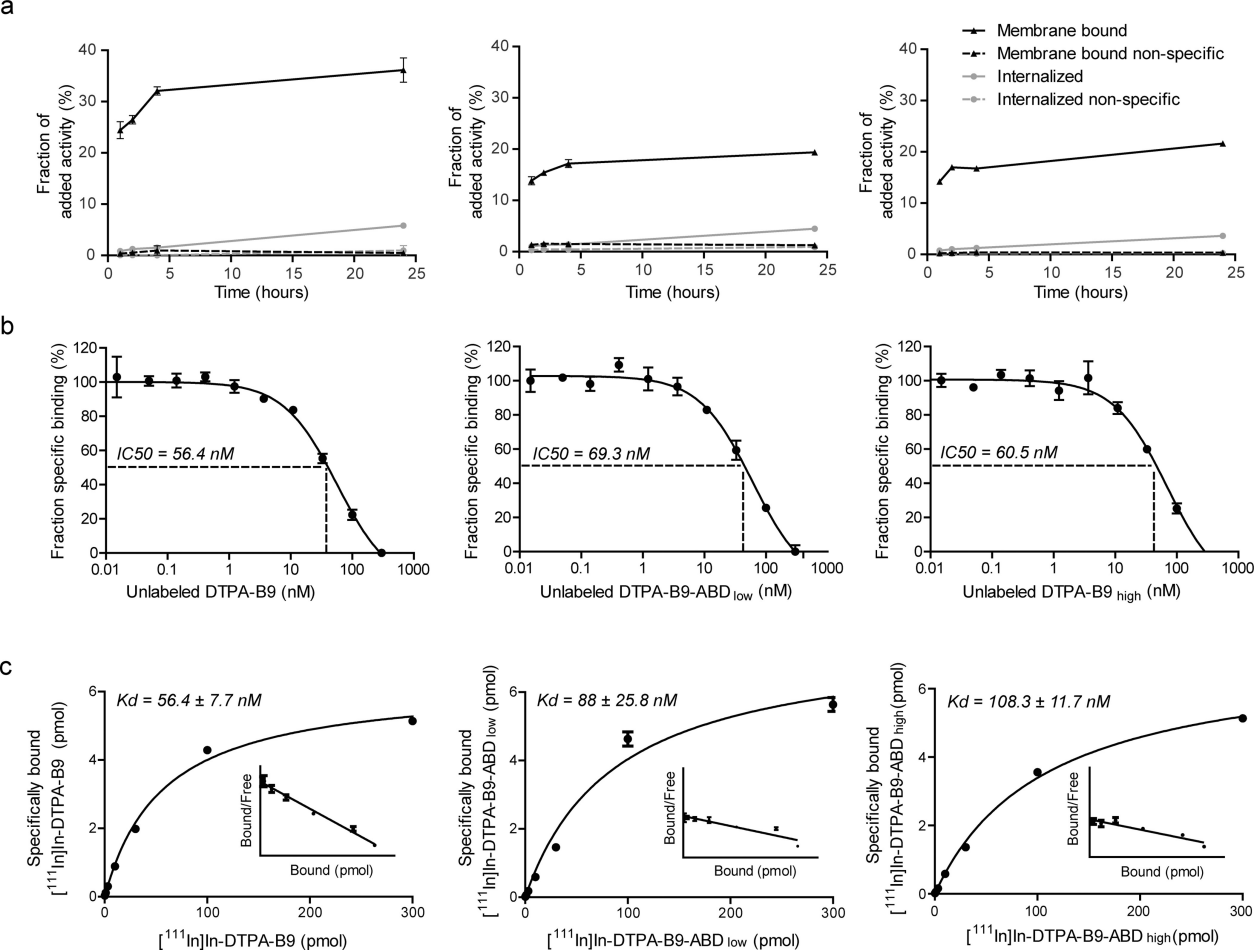
*In vitro* characterization with SKRC-52 cells showing (A) internalization and binding, (B) IC_50_ analysis, and (C) specific binding and scatchard curves of [^111^In]In-DTPA-B9 (left), [^111^In]In-DTPA-B9-ABD_low_ (middle), and [^111^In]In-DTPA-B9-ABD_high_ (right).

[^111^In]In-DTPA-B9, [^111^In]In-DTPA-B9-ABD_low_, and [^111^In]In-DTPA-B9-ABD_high_ bound to SK-RC-52 cells in a CAIX specific manner, as demonstrated by the absence of binding in the presence of an excess of unlabeled B9. The internalization rate of all tracers was low (Figure 2a). Half maximal inhibitory concentrations (IC_50_) of DTPA-B9, DTPA-B9-ABD_low_, and DTPA-B9-ABD_high_ were 56.4, 69.3, and 60.5, respectively (Figure 2b). In a Scatchard analysis for a single binding site, the dissociation constants of [^111^In]In-DTPA-B9, [^111^In]In-DTPA-B9-ABD_low_, and [^111^In]In-DTPA-B9-ABD_high_ were 56.4 ± 7.7 nM, 88 ± 25.8 nM, and 108 ± 12 nM, respectively (Figure 2c).

### [^111^In]In-DTPA-B9 Accumulates Specifically in CAIX Positive Xenografts

**Figure 3:**
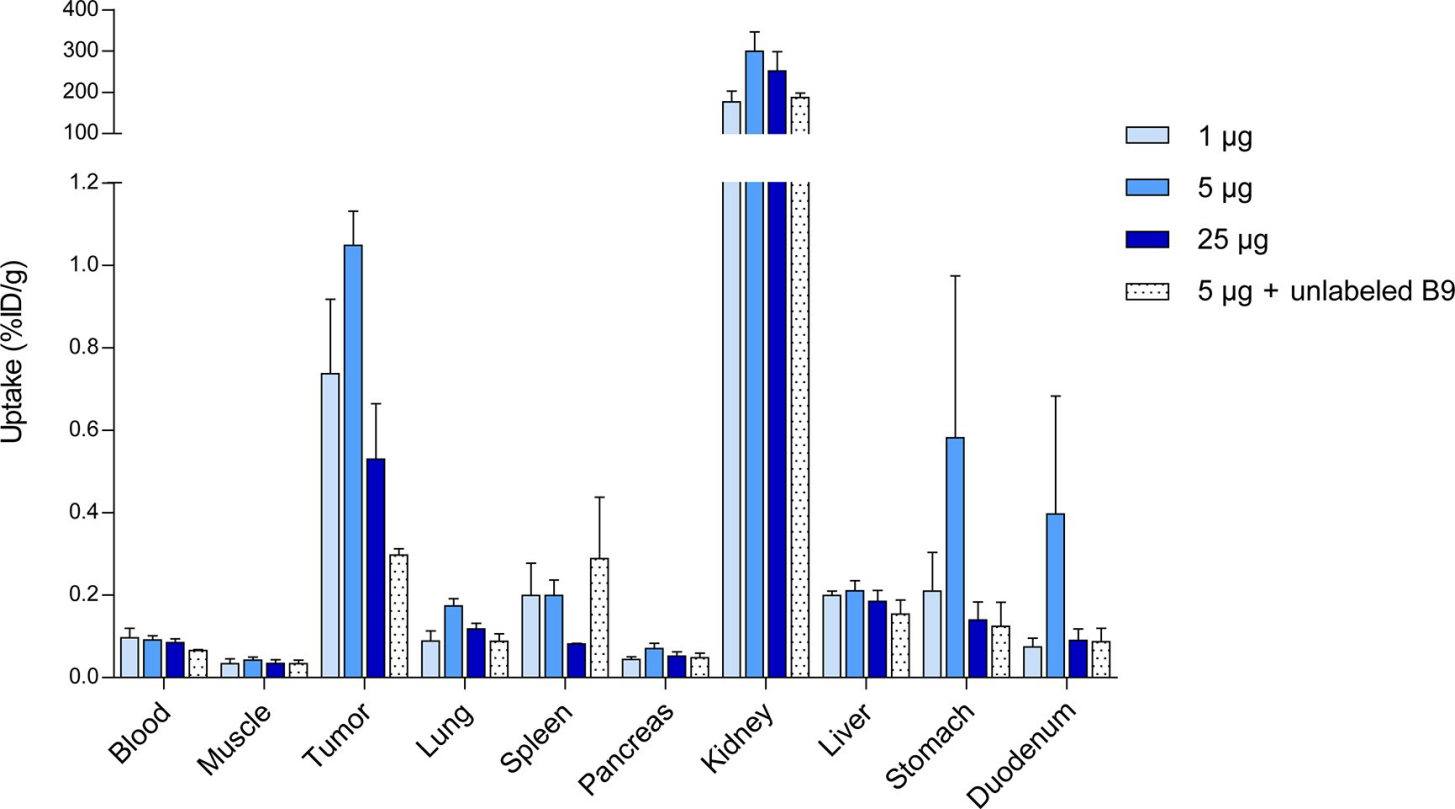
Biodistribution analyses showing the percentage of the injected dose per gram of tissue (%ID/g) at 4 h upon intravenous injection of various protein doses of [^111^In]In-DTPA-B9, with or without preinjection of 300 μg of unlabeled B9.

A dose-escalation experiment was done with [^111^In]In-DTPA-B9 to determine the optimal tracer dose in the SCCNij153 tumor model. A maximum relative tumor uptake of 1.05 ± 0.14%ID/g was found at 4 h after injection of 5 μg [^111^In]In-DTPA-B9, with tumor-to-blood and tumor-to-muscle ratios 11.96 ± 3.51 and 26.91 ± 11.43, respectively (Figure 3 and Supplementary Table 1). The significant decrease in tumor uptake of [^111^In]In-DTPA-B9 upon injection of unlabeled B9 (0.30 ± 0.03%ID/g, *p* = 0.0009) indicated specificity for CAIX.

### Fusion with Albumin-Binding Domains Prolong Circulation Time and Increase Tumor Uptake of [^111^In]In-DTPA-B9

**Figure 4:**
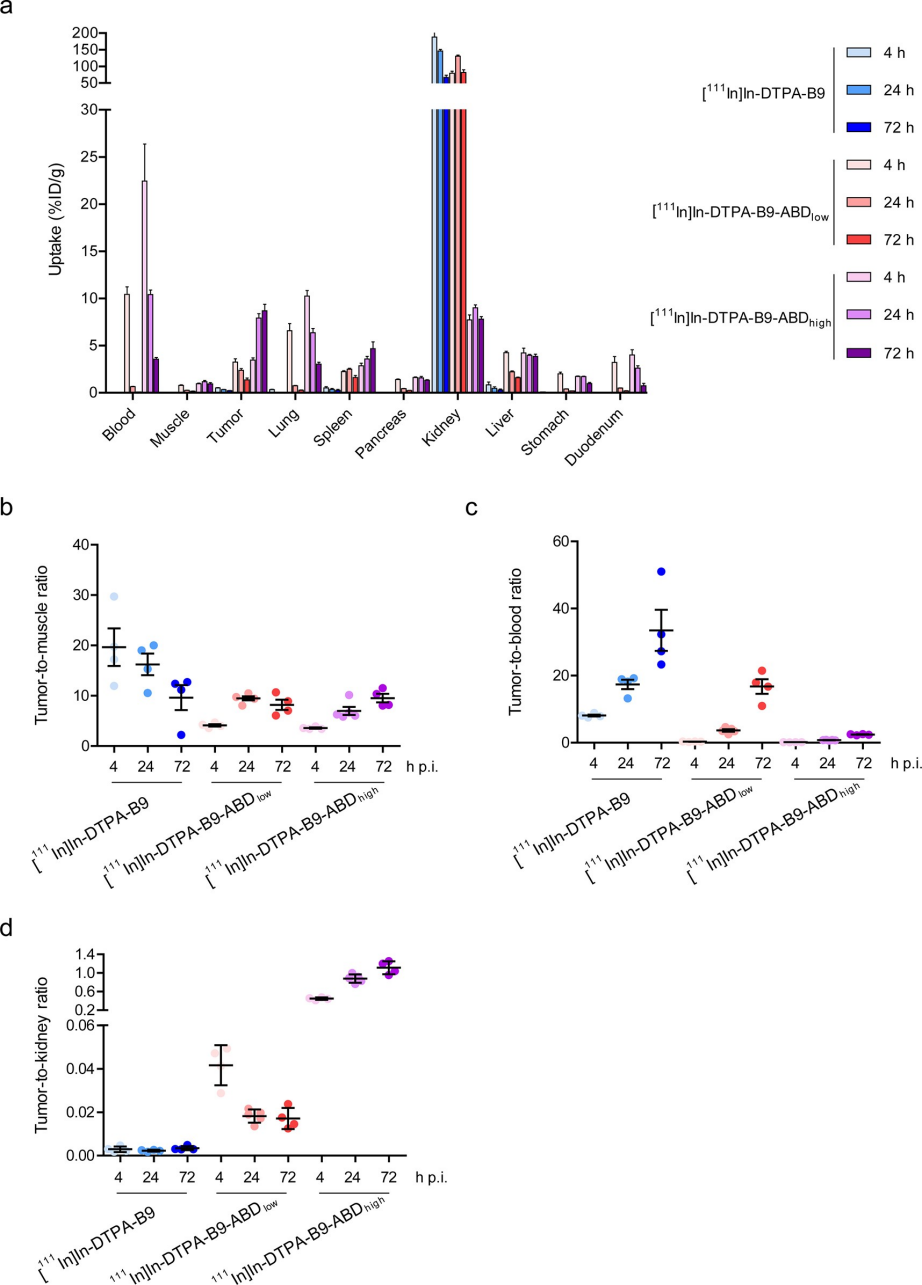
(A) Biodistribution analysis of [^111^In]In-DTPA-B9 and [^111^In]In-DTPA-B9-ABD_low_ and [^111^In]In-DTPA-B9-ABD_high_ showing (A) the percentage of injected dose per gram of tissue (%ID/g) at 4, 24, and 72 h after intravenous injection (B) tumor-to-muscle, (C) tumor-to-blood, and (D) tumor-to-kidney ratios.

To prolong plasma half-life and increase tumor uptake of radiolabeled B9, it was expressed in fusion with an albumin-binding domain with varying affinity, ABD_low_ or ABD_high_. Biodistribution was compared at 4, 24, and 72 h post injection (Figure 4a and Supplementary Table 2). The addition of ABD_low_ and ABD_high_ to B9 led to increased tumor uptake; however, enhanced blood retention led to lower tumor-to-blood ratios when compared to B9. Maximal tumor-to-muscle and acceptable tumor-to-blood ratios (>2 for application in molecular imaging) were reached at 4, 24, and 72 h post injection for [^111^In]In-DTPA-B9, [^111^In]In-DTPA-B9-ABD_low_, and [^111^In]In-DTPA-B9-ABD_high_, respectively (Figure 4b,c and Supplementary Table 2). Fusion of B9 to ABD_high_ led to a strong decrease in kidney uptake, which was reflected in increased tumor-to-kidney ratios at all time points, while fusion to ABD_low_ led to a lower renal uptake at 4 h post injection only (Figure 4a,d and Supplementary Table 2).

### Increased Tumor Uptake of B9-ABD Variants Is Partly CAIX Independent

**Figure 5:**
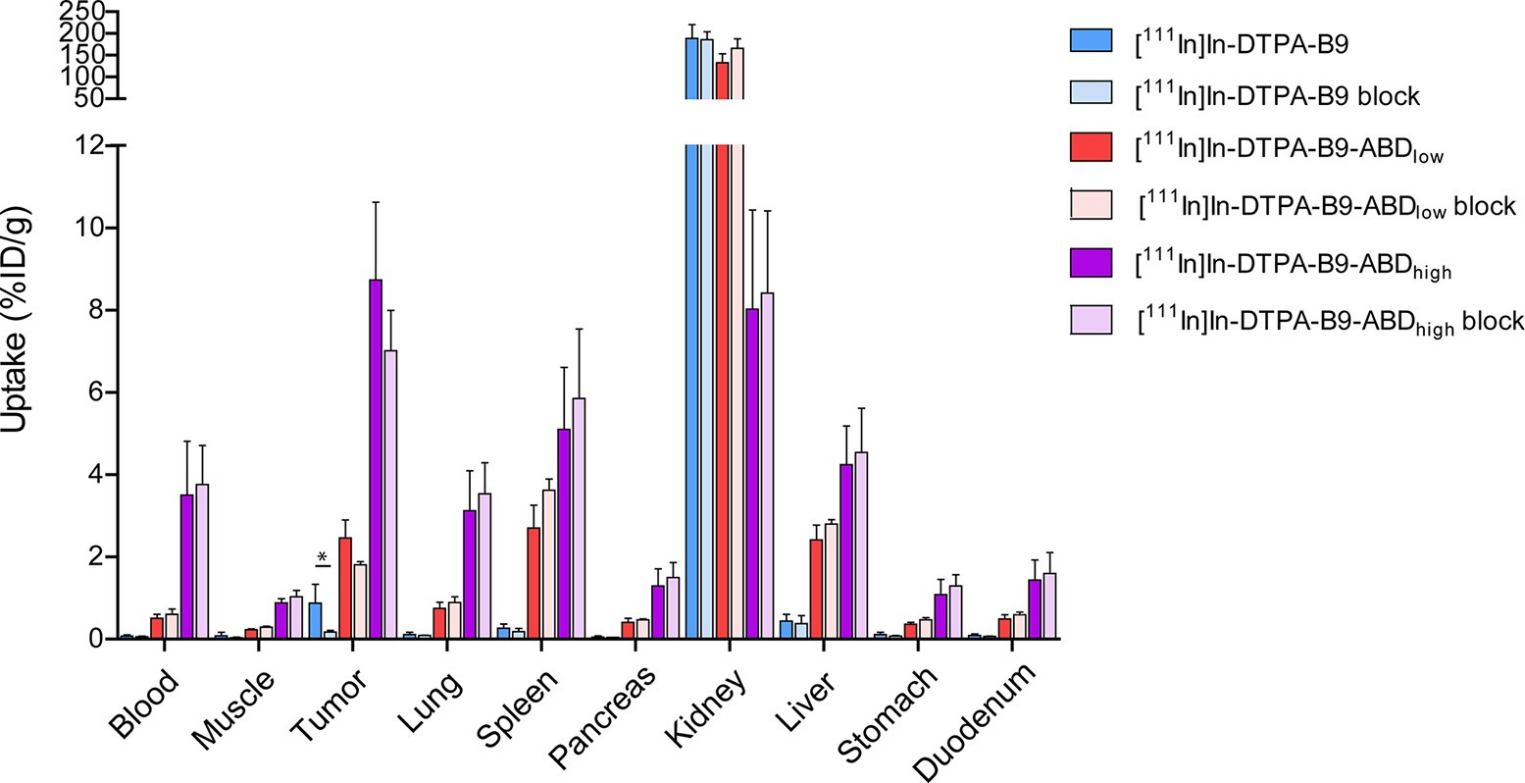
Biodistribution analysis of [^111^In]In-DTPA-B9 (4 h after injection), [^111^In]In-DTPA-B9-ABD_low_ (24 h post injection), and [^111^In]In-DTPA-B9-ABD_high_ (72 h post injection) with or without injection of a molar excess girentuximab at 24 h before tracer injection.

**Figure 6:**
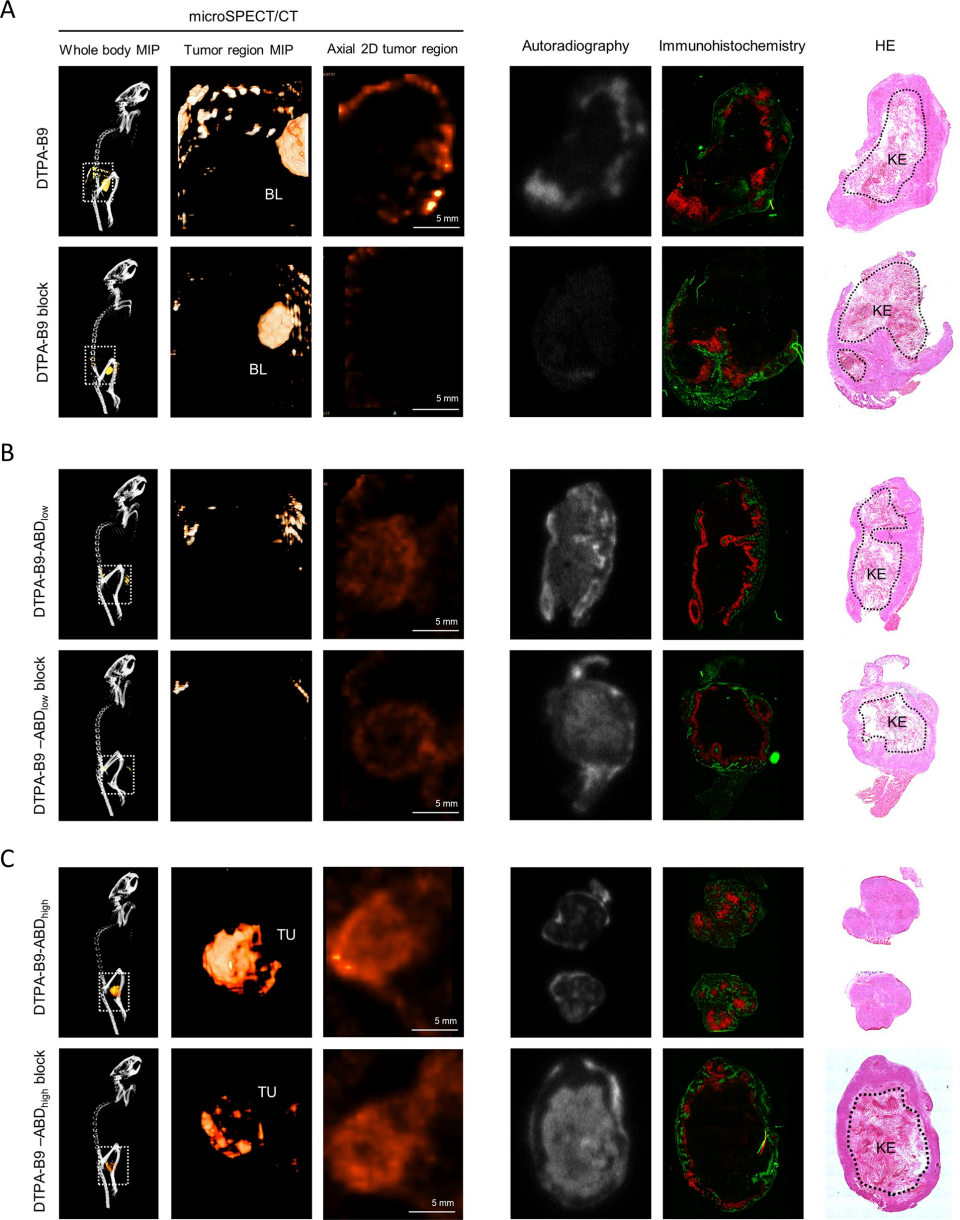
Accumulation of (A) [^111^In]In-DTPA-B9 with and without excess girentuximab (block) at 4 h, (B) [^111^In]In-DTPA-B9-ABD_low_ with and without block at 24 h, and (C) [^111^In]In-DTPA-B9-ABD_high_ with and without block at 72 h, as visualized with microSPECT/CT (left panels, lateral whole body MIP, lateral tumor region MIP and axial 2D scan of the tumor region). Note that we only scanned the tumor area for 2 h; this area is indicated with the dotted rectangle in the whole body SPECT images. In the right panel, autoradiography, an immunohistochemistry (IHC) image showing staining for CAIX (red) and tissue perfusion with Hoechst (green) and an HE image are shown. (BL = bladder, TU = tumor, KE = keratinized tumor regions as indicated with circumscribed areas in the HE images).

To determine CAIX specificity of uptake, mice were injected with unlabeled monoclonal anti-CAIX antibody girentuximab 24 h before administration of ^111^In-labeled B9-tracers. Girentuximab competed effectively with binding of [^111^In]In-DTPA-B9 to SK-RC-52 cells *in vitro* (Supplementary Figure 1), showing that both molecules bind similar epitopes of CAIX. *Ex vivo* biodistribution studies were performed at 4, 24, and 72 h after injection of [^111^In]In-DTPA-B9, [^111^In]In-DTPA-B9-ABD_low_, and [^111^In]In-DTPA-B9-ABD_high_, respectively (Figure 5 and Supplementary Table 3). Tumor uptake of [^111^In]In-DTPA-B9 was almost completely inhibited (*p* = 0.05) upon injection of unlabeled girentuximab, but incomplete reduction of tumor uptake was observed for [^111^In]In-DTPA-B9-ABD_low_ (*p* = 0.06) and [^111^In]In-DTPA-B9-ABD_high_ (*p* = 0.21). Furthermore, microSPECT images of the tumor regions were acquired and registered to a whole-body CT scan (one representative scan in Figure 6a, and additional scans in Supplementary Figures 2–4). Tumor uptake of [^111^In]In-DTPA-B9 at 4 h could not be visualized, and only bladder uptake was observed (Figure 6a). For [^111^In]In-DTPA-B9-ABD_low_ (Figure 6b) and [^111^In]In-DTPA-B9-ABD_high_ (Figure 6c), heterogeneous uptake in the SCCNij153 xenograft could be visualized, which partly remained upon injection of an excess of girentuximab.

Signal of autoradiography in excised tumor sections correlated positively with CAIX immunohistochemistry for all B9-based tracers (Supplementary Figure 5a–d). Importantly, these analyses were done on annotated vital tumor areas, excluding regions of keratinization in the tumor centers (marked as KE in Figure 6). The signal on autoradiography was efficiently blocked in CAIX positive regions but remained in the CAIX-negative keratinized regions for the blocked groups of B9-ABD_low_ and B9-ABD_high_ (Figure 6b,c).

## Discussion

Molecular imaging of hypoxic regions in solid tumors has the potential to steer treatment regimens. In the present study, we have developed and characterized novel radiotracers for SPECT imaging of the endogenous hypoxia-related marker CAIX, based on VHH B9.^31^

B9-variants were site-specifically equipped with maleimide-DTPA. We showed moderate binding affinity of conjugated B9 to CAIX-expressing SK-RC-52 cells, which was not affected by expression in fusion with either ABD_low_ or ABD_high_. *In vivo*, longer plasma residence led to increased tumor uptake in SCCNij153 xenografts, and acceptable tumor-to-blood ratios (>2 for application in molecular imaging) were reached at later time points post injection for [^111^In]In-DTPA-B9-ABD_low_ and [^111^In]In-DTPA-B9-ABD_high_ when compared to [^111^In]In-DTPA-B9_._ Uptake of [^111^In]In-DTPA-B9-ABD_low_ at 24 h and [^111^In]In-DTPA-B9-ABD_high_ at 72 h could be visualized with SPECT imaging. Since relatively high levels of [^111^In]In-DTPA-B9-ABD_high_ were found in the blood at 72 h, a later time point after injection could be more optimal for imaging, provided that tumor uptake of the tracer is retained. This would, however, eliminate the advantage of early imaging with VHHs when compared to imaging with monoclonal antibodies, which is also performed at 3–5 days post injection.^35^ Importantly, the lower tumor accumulation of B9 when compared to that of labeled antibody girentixumab in other studies can be attributed to the 10-fold lower affinity and the inefficient internalization when compared to girentuximab.^36^

Increased tumor uptake of both B9-ABD tracers could not efficiently be blocked by administration of unlabeled girentuximab, which binds a similar epitope of CAIX, indicating that the uptake is partly CAIX-independent. Autoradiography analysis indeed confirmed tracer uptake of ABD-conjugates in CAIX-negative keratinized tumor tissue, hypothetically due to increased residence of plasma proteins, possibly including albumin in this tissue type. Importantly, keratinization is a common histological feature of squamous cell carcinomas;^37^ thus, aspecific uptake in these areas could restrict the application of these tracers in hypoxia quantification. Specific tumor accumulation using VHHs provided with an ABD might be possible for other tumor types lacking keratinized tissue, which remains to be investigated. Also, other groups have reported increased tumor uptake of peptide-, affibody-, and nanobody-based tracers conjugated to a variety of ABDs. In some studies, target specificity of the tracer was confirmed by lack of uptake in antigen-blocked groups or in nontarget expressing tumors,^29^ while, in other studies, residual uptake in blocked groups or nontarget expressing tumors was observed.^28^ Depending on the application, the current study underscores the importance of investigating the specificity of the tracer uptake.

Low absolute uptake of B9 precludes the use of this tracer for imaging of low-abundant targets such as CAIX in hypoxic tumors. The addition of ABD increases absolute but also nonspecific tumor uptake, also eliminating the possibility of using these constructs for precise localization and quantification of hypoxic tumor regions. Alternatively, other clearance affecting approaches could be used such as PEGylation, fusion to Fc domains, or multivalent constructs.^15^ Aside from imaging, the addition of ABD could also be employed to increase absolute tumor uptake and decrease renal uptake of small tracers equipped with therapeutic radionuclides, thereby increasing therapeutic efficacy and decreasing nephrotoxicity. We observed a strong decrease in renal accumulation, especially for the ABD_high_ conjugate. This might reflect the high affinity association of the tracer with plasma albumin that lasts for up to 72 h and induces increased hepatic excretion, while B9-ABD_low_ has dissociated from plasma albumin within 24 h, and can be removed through glomerular filtration.

In recent years, multiple CAIX-targeting radiotracers have been studied.^38^ An important advantage of VHHs as imaging tracers is their modular character. They can be site-specifically modified, thereby controlling target binding activity and biodistribution. Furthermore, they can be easily adjusted in size and valency and expressed in fusion with other proteins.

## Conclusions

We demonstrated that the developed B9 tracers have a moderate affinity to CAIX *in vitro* and that they target head and neck cancer xenografts *in vivo.* This targeting was entirely CAIX specific for native B9, and although the addition of ABDs increased plasma residence and tumor uptake, tumor uptake of the ABD-modified VHH was partly CAIX-independent in keratinized areas. The addition of an ABD to B9, therefore, did not improve SPECT imaging contrast of CAIX-expressing regions for hypoxia quantification in head and neck cancer. This strategy could, however, be used to increase the absolute tumor uptake and to optimize tumor-to-kidney ratios for therapeutic applications of VHHs.

## Supporting Information Available

Uptake values and tumor-to-organ ratios of the various tracers in the biodistribution experiments, SPECT/CT images, autoradiography and immunohistochemistry figures of all animals included in the study, and the analyses of spatial correlation of autoradiography and CAIX expression on immunohistochemistry (PDF)
