# Supplementary material for: Novel VHH-Based Tracers with Variable Plasma Half-Lives for Imaging of CAIX-Expressing Hypoxic Tumor Cells
Source: Mol Pharm. 2022 Jan 19;19(10):3511–20. doi: 10.1021/acs.molpharmaceut.1c00841 (PMC9533306; doi:10.1021/acs.molpharmaceut.1c00841)
Supplement: Supplementary file 1 — mp1c00841_si_004.pdf [file mp1c00841_si_004.pdf]

Supporting information for

Novel VHH-based tracers with variable plasma half-lives for imaging of CAIX-expressing hypoxic tumor cells

Sanne A.M. van Lith[1#*], Fokko J. Huizing[1,2#], Gerben M. Franssen[1], Bianca A.W. Hoeben[2,3], Jasper Lok[2], Sofia Doulkeridou[4], Otto C. Boerman[1], Martin Gotthardt[1], Paul M.P. van Bergen en Henegouwen[4], Johan Bussink[2], Sandra Heskamp[1]

# Authors contributed equally

[1]Department of Medical Imaging, Radboud University Medical Center, Nijmegen, 6500 HB, The Netherlands

[2]Department of Radiation Oncology, Radboud University Medical Center, Nijmegen, 6500 HB, The Netherlands

[3]Department of Radiation Oncology, University Medical Center Utrecht, Utrecht, 3508 GA, The Netherlands

[4]Department of Cell Biology, University of Utrecht, Utrecht, 3584 GE, The Netherlands

* Corresponding author:

Sanne A.M. van Lith, PhD

ORCID ID: 0000-0001-8584-2546

Radboud University Medical Center, Department of Medical Imaging

Geert Grooteplein zuid 10

6525 GA Nijmegen

The Netherlands

Sanne.vanLith@radboudumc.nl

Table S1 Uptake of various protein doses of B9-DTPA in tissues at 4 hours post injection expressed as mean ± SD %ID/g and tumor-to-normal ratios

	B9<br>1 µg (n=5)	B9<br>5 µg (n=3)	B9<br>25 µg (n=3)	B9<br>5 µg + unlabeled B9 (n=3)
**Biodistribution**				
Blood	0.10 ± 0.05	0.09 ± 0.02	0.09 ± 0.02	0.07 ± 0.00
Muscle	0.03 ± 0.03	0.04 ± 0.01	0.03 ± 0.02	0.03 ± 0.01
Tumor	0.74 ± 0.40	1.05 ± 0.14	0.53 ± 0.23	0.30 ± 0.03
Lung	0.09 ± 0.06	0.17 ± 0.03	0.12 ± 0.02	0.09 ± 0.03
Spleen	0.20 ± 0.18	0.20 ± 0.06	0.08 ± 0.002	0.29 ± 0.26
Pancreas	0.04 ± 0.01	0.07 ± 0.02	0.05 ± 0.02	0.05 ± 0.02
Kidney	176.98 ± 58.95	299.03 ± 81.68	251.70 ± 80.58	188.94 ± 20.01
Liver	0.20 ± 0.02	0.21 ± 0.04	0.18 ± 0.05	0.15 ± 0.06
Stomach	0.21 ± 0.21	0.58 ± 0.68	0.14 ± 0.08	0.12 ± 0.10
Duodenum	0.07 ± 0.05	0.40 ± 0.20	0.09 ± 0.05	0.09 ± 0.06
**Tumor-to-normal ratios**				
Tumor-to-muscle	25.80 ± 7.38	26.91 ± 11.43	15.73 ± 3.04	9.95 ± 3.96
Tumor-to-blood	7.86 ± 3.27	11.96 ± 3.51	6.08 ± 2.17	4.52 ± 0.45
Tumor-to-kidney	0.004 ± 0.002	0.004 ± 0.001	0.002 ± 0.001	0.002 ± 0.0001

Table S2 Uptake of B9 tracers in tissues at 4, 24 or 72 hours post injection expressed as mean ± SD %ID/g and tumor-to-normal ratios

	B9 4 hrs (n=4)	B9 24 hrs (n=4)	B9 72 hrs (n=4)	B9-ABD$_{low}$ 4 hrs (n=4)	B9-ABD$_{low}$ 24 hrs (n=5)	B9-ABD$_{low}$ 72 hrs (n=4)	B9-ABD$_{high}$ 4 hrs (n=4)	B9-ABD$_{high}$ 24 hrs (n=5)	B9-ABD$_{high}$ 72 hrs (n=4)
**Biodistribution**									
Blood	0.06 ± 0.01	0.02 ± 0.001	0.01 ± 0.001	10.44 ± 1.59	0.65 ± 0.07	0.08 ± 0.01	22.44 ± 7.89	10.53 ± 1.09	3.57 ± 0.35
Muscle	0.03 ± 0.01	0.02 ± 0.01	0.04 ± 0.04	0.78 ± 0.11	0.24 ± 0.05	0.17 ± 0.02	0.97 ± 0.11	1.15 ± 0.28	0.95 ± 0.27
Tumor	0.51 ± 0.08	0.33 ± 0.04	0.22 ± 0.05	3.26 ± 0.69	2.39 ± 0.44	1.37 ± 0.37	3.46 ± 0.48	8.02 ± 1.04	8.70 ± 1.34
Lung	0.33 ± 0.1	0.05 ± 0.004	0.03 ± 0.002	6.59 ± 1.53	0.74 ± 0.07	0.28 ± 0.02	10.25 ± 1.19	6.66 ± 0.99	3.05 ± 0.39
Spleen	0.51 ± 0.29	0.37 ± 0.19	0.26 ± 0.20	2.25 ± 0.21	2.56 ± 0.20	1.62 ± 0.45	2.87 ± 0.55	3.61 ± 0.62	4.68 ± 1.41
Pancreas	0.04 ± 0.003	0.03 ± 0.002	0.03 ± 0.004	1.14 ± 0.13	0.45 ± 0.03	0.25 ± 0.03	1.63 ± 0.08	1.55 ± 0.36	1.32 ± 0.12
Kidney	188.36 ± 48.08	146.5 ± 9.04	66.57 ± 14.15	79.36 ± 12.55	133.71 ± 7.11	81.45 ± 16.80	7.72 ± 1.07	9.13 ± 0.67	7.80 ± 0.54
Liver	0.86 ± 0.57	0.49 ± 0.29	0.3 ± 0.19	4.25 ± 0.30	2.23 ± 0.25	1.61 ± 0.10	4.23 ± 1.02	3.98 ± 0.22	3.85 ± 0.48
Stomach	0.08 ± 0.01	0.05 ± 0.01	0.03 ± 0.01	2.02 ± 0.35	0.39 ± 0.04	0.20 ± 0.02	1.74 ± 0.05	1.75 ± 0.10	0.99 ± 0.23
Duodenum	0.07 ± 0.003	0.05 ± 0.01	0.02 ± 0.003	3.22 ± 1.24	0.51 ± 0.03	0.19 ± 0.05	4.02 ± 1.06	2.67 ± 0.55	0.75 ± 0.51
**Tumor-to-normal ratios**									
Tumor-to-muscle	19.66 ± 7.45	16.23 ± 4.29	9.62 ± 4.97	4.13 ± 0.46	9.49 ± 0.89	8.19 ± 2.03	3.59 ± 0.24	6.99 ± 1.78	9.52 ± 1.69
Tumor-to-blood	8.10 ± 0.58	17.39 ± 2.82	33.49 ± 12.27	0.31 ± 0.06	3.66 ± 0.81	16.77 ± 4.36	0.16 ± 0.04	0.76 ± 0.09	2.43 ± 0.15
Tumor-to-kidney	0.003 ± 0.001	0.002 ± 0.0004	0.003 ± 0.001	0.042 ± 0.009	0.018 ± 0.003	0.017 ± 0.005	0.449 ± 0.026	0.879 ± 0.090	1.113 ± 0.139

Table S3 Uptake of B9 tracers in tissues with or without injection of unlabeled girentuximab (blocked) expressed as mean ± SD %ID/g

	B9 (n=4, 4 hours post injection)	B9 Blocked (n=3, 4 hours post injection)	B9-ABD_{low} (n=3, 24 hours post injection)	B9-ABD_{low} Blocked (n=3, 24 hours post injection)	B9-ABD_{high} (n=4, 72 hours post injection)	B9-ABD_{high} Blocked (n=3, 72 hours post injection)
**Biodistribution**						
Blood	0.08 ± 0.03	0.06 ± 0.01	0.51 ± 0.09	0.6 ± 0.13	3.50 ± 1.31	3.76 ± 0.95
Muscle	0.08 ± 0.09	0.04 ± 0.02	0.23 ± 0.02	0.29 ± 0.02	0.88 ± 0.10	1.03 ± 0.16
Tumor	0.87 ± 0.46	0.17 ± 0.04	2.46 ± 0.44	1.81 ± 0.08	8.73 ± 1.89	7.01 ± 0.98
Lung	0.11 ± 0.06	0.09 ± 0.01	0.75 ± 0.14	0.89 ± 0.14	3.12 ± 0.97	3.53 ± 0.76
Spleen	0.26 ± 0.11	0.18 ± 0.08	2.71 ± 0.55	3.62 ± 0.27	5.10 ± 1.51	5.86 ± 1.68
Pancreas	0.05 ± 0.03	0.04 ± 0.01	0.41 ± 0.10	0.47 ± 0.02	1.29 ± 0.41	1.50 ± 0.36
Kidney	188.43 ± 31.96	185.8 ± 18.15	132.81 ± 20.35	165.49 ± 22.68	8.02 ± 2.41	8.41 ± 1.99
Liver	0.44 ± 0.16	0.37 ± 0.20	2.42 ± 0.36	2.79 ± 0.11	4.24 ± 0.95	4.54 ± 1.07
Stomach	0.11 ± 0.06	0.07 ± 0.02	0.36 ± 0.05	0.47 ± 0.05	1.09 ± 0.36	1.29 ± 0.27
Duodenum	0.09 ± 0.04	0.07 ± 0.01	0.49 ± 0.10	0.60 ± 0.06	1.43 ± 0.49	1.60 ± 0.51

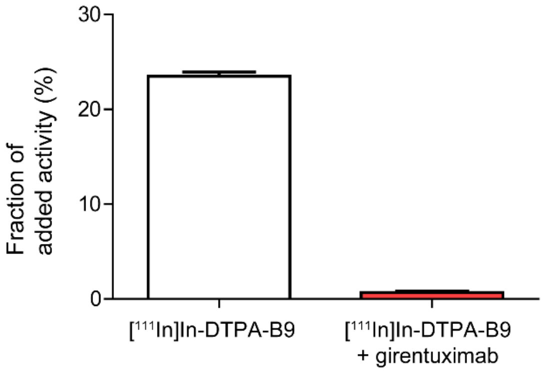

Fig. S1 Binding of [^{111}In]In-DTPA-B9 to SKRC-52 cells in absence and presence of an excess unlabeled monoclonal anti-CAIX antibody girentuximab

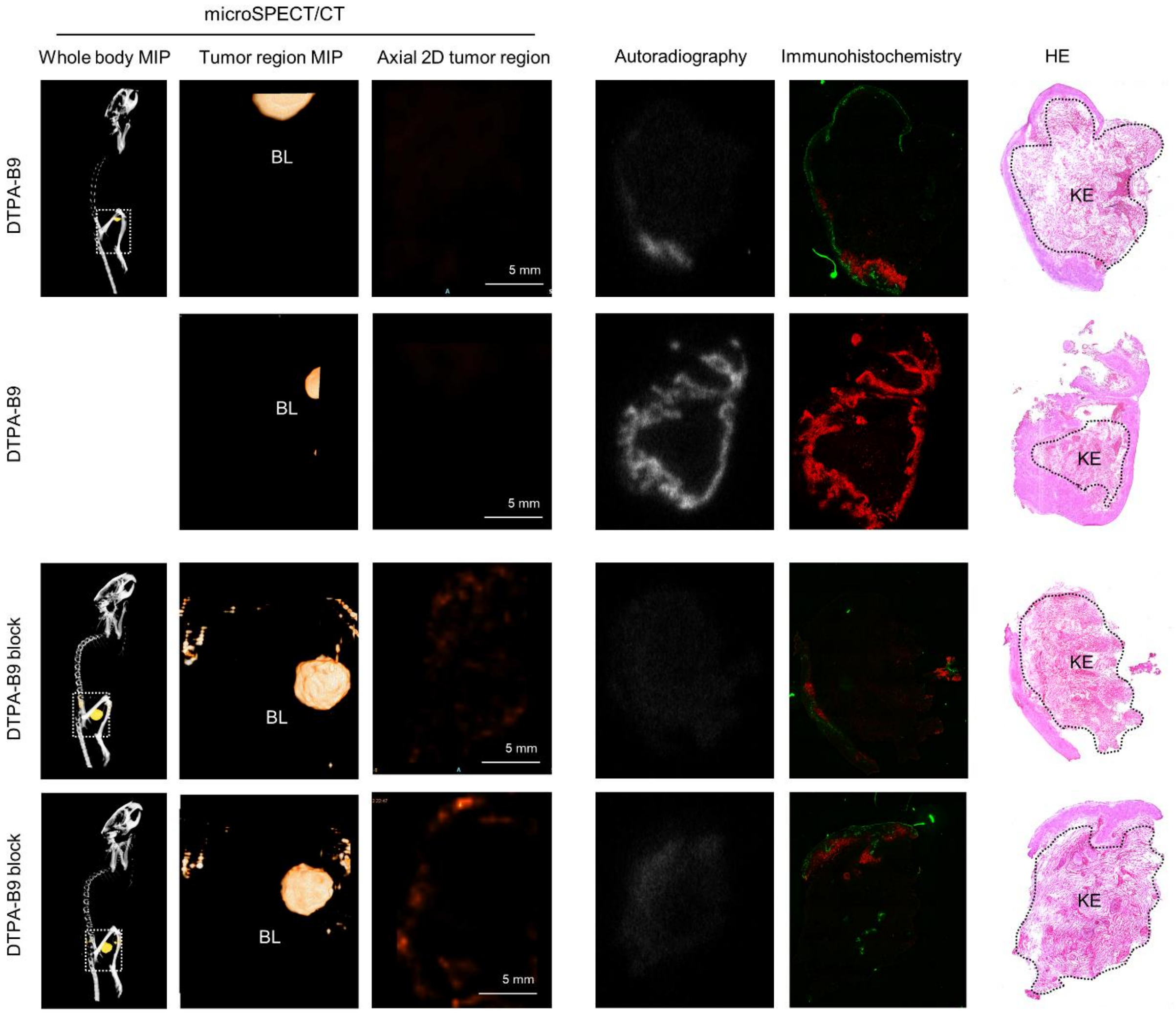

Fig. S2 Accumulation of [^{111}In]In-DTPA-B9 with and without excess girentuximab (block), as visualized with microSPECT/CT (left panels, lateral whole body MIP, lateral tumor region MIP and axial 2D scan of the tumor region). Note that we only scanned the tumor area for 2 hours, this area is indicated with the dotted lines in the whole body SPECT image. Since CT imaging failed in one mouse, only the SPECT scan of the tumor area is shown in row 2. In the right panel, autoradiography, an immunohistochemistry (IHC) image showing staining for CAIX (red) and tissue perfusion with Hoechst (green) and an HE image are shown. (BL = bladder, KE = keratinized tumor regions as indicated with dotted lines in the HE image)

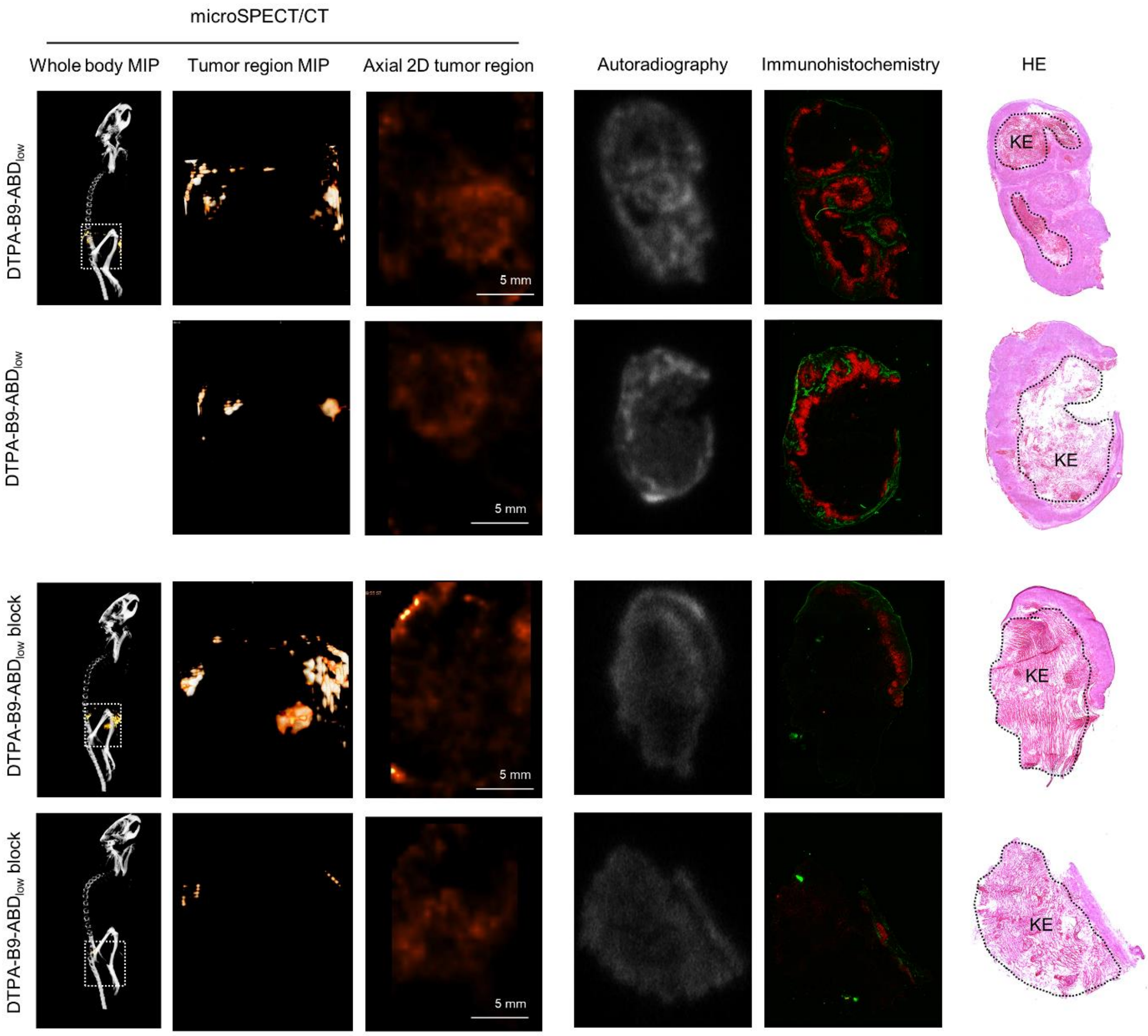

Fig. S3 Accumulation of [^{111}In]In-DTPA-B9-ABD$_{low}$ with and without excess unlabeled girentuximab (block), as visualized with microSPECT/CT (left panels, lateral whole body MIP, lateral tumor region MIP and axial 2D scan of the tumor region). Note that we only scanned the tumor area for 2 hours, this area is indicated with the dotted lines in the whole body SPECT image. Since CT imaging failed in one mouse, only the SPECT scan of the tumor area is shown in row 2. In the right panel, autoradiography, an immunohistochemistry (IHC) image showing staining for CAIX (red) and tissue perfusion with Hoechst (green) and an HE image are shown. (KE = keratinized tumor regions as indicated with dotted lines in the HE images)

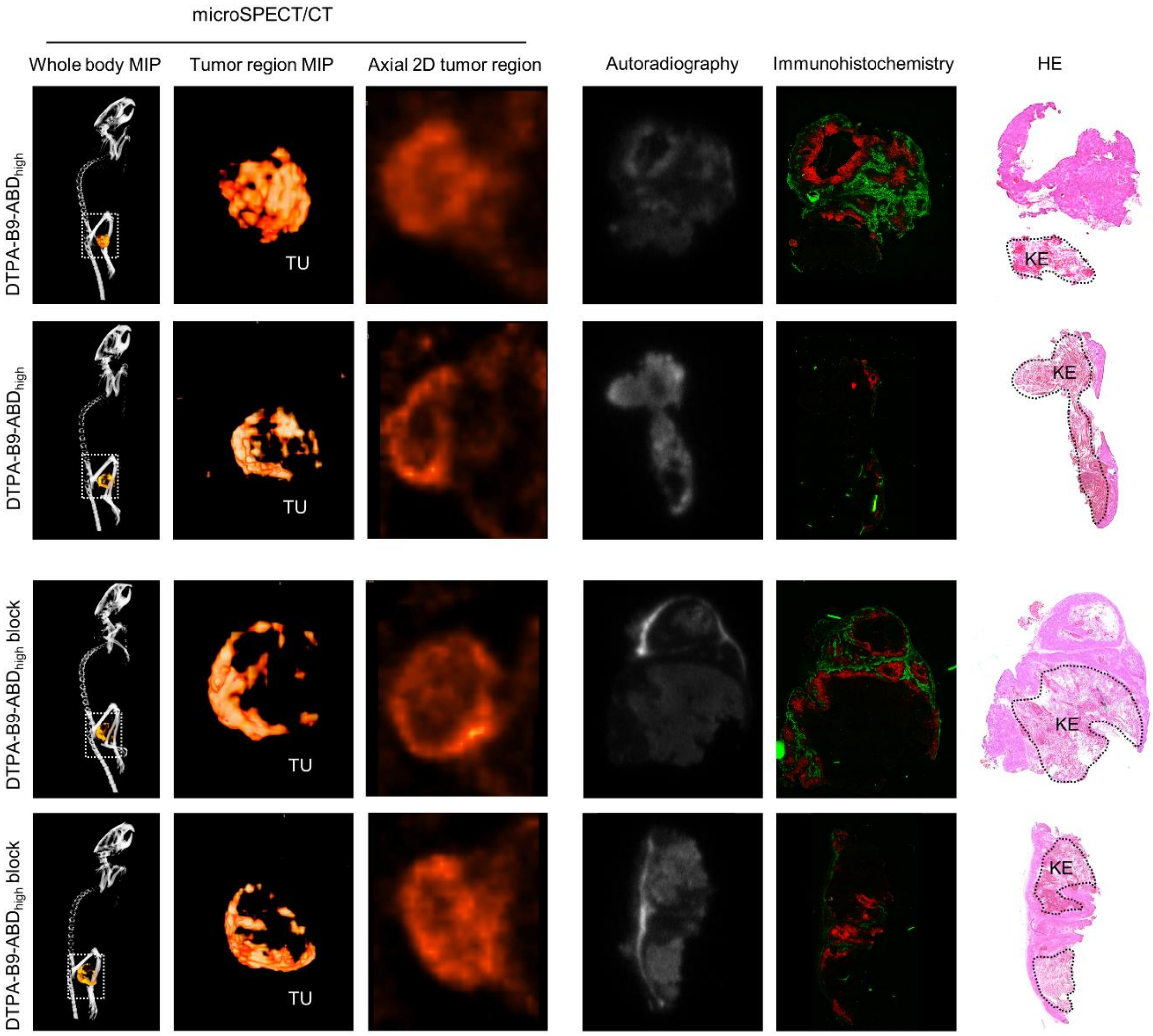

Fig. S4 Accumulation of [^{111}In]In-DTPA-B9-ABD$_{high}$ with and without excess unlabeled girentuximab (block), as visualized with microSPECT/CT (left panels, lateral whole body MIP, lateral tumor region MIP and 2D scan). Note that we only scanned the tumor area for 2 hours, this area is indicated with the dotted lines in the whole body SPECT image. In the right panel, autoradiography, an immunohistochemistry (IHC) image showing staining for CAIX (red) and tissue perfusion with Hoechst (green) and an HE image are shown. (KE = keratinized tumor regions as indicated with dotted lines in the HE image)

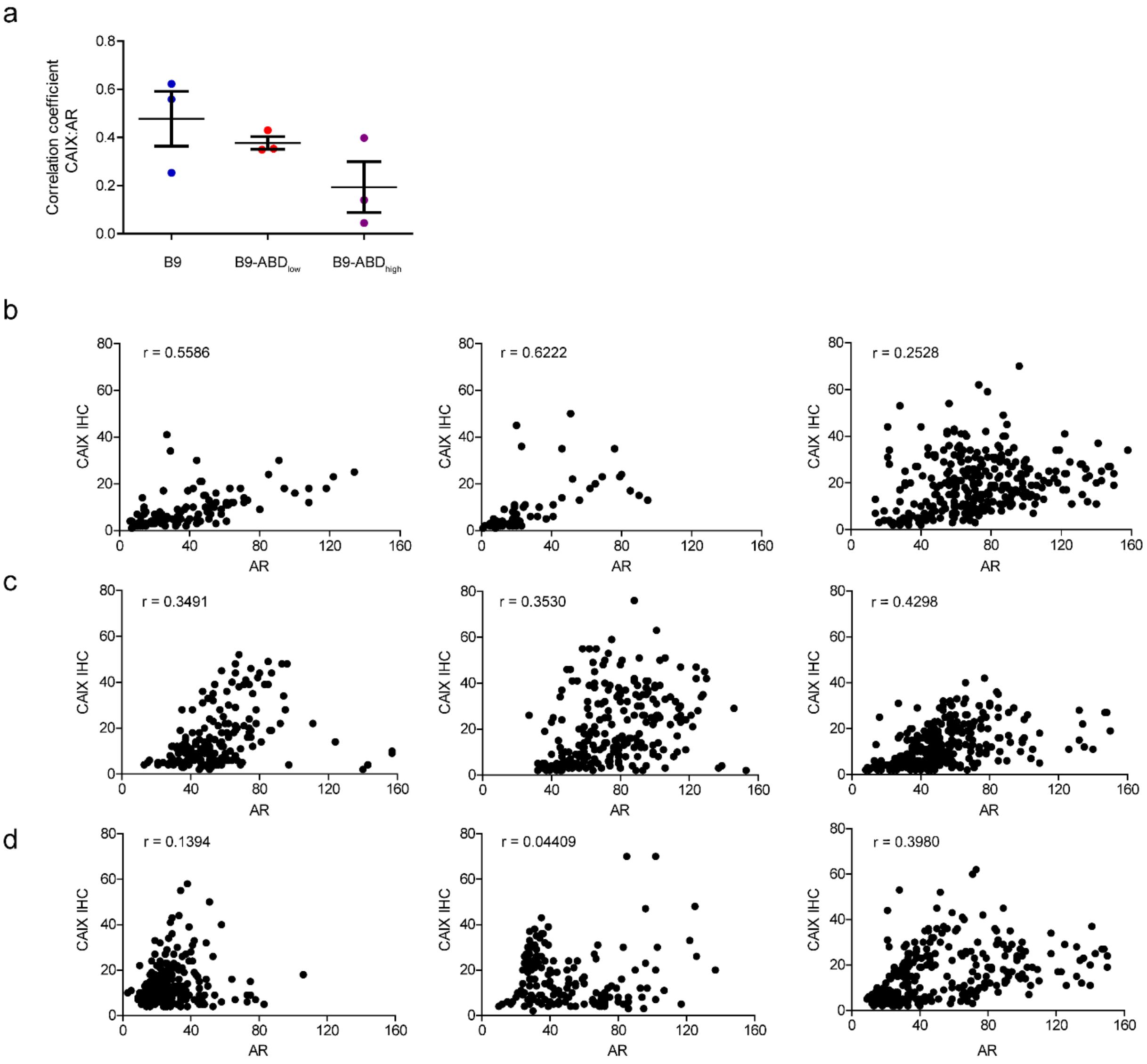

Fig. S5 a) Pearsons correlation coefficients as determined in a colocalization analysis of signals on autoradiography and CAIX immunohistochemistry of SCCNij153 tumor sections of mice injected with either of the three B9-tracers b) correlation of autoradiography and CAIX immunohistochemistry images of tumors of 3 mice injected with [^{111}In]In-DTPA-B9 c) correlation of autoradiography and CAIX immunohistochemistry images of tumors of 3 mice injected with [^{111}In]In-DTPA-B9 [^{111}In]In-DTPA-B9-ABD$_{low}$ and d) correlation of autoradiography and CAIX immunohistochemistry images of tumors of 3 mice injected with [^{111}In]In-DTPA-B9 [^{111}In]In-DTPA-B9-ABD$_{hig}$
